# Carbon-based molecular qubits: a chemical pathway to ambient quantum technologies

**DOI:** 10.1093/nsr/nwag129

**Published:** 2026-03-06

**Authors:** Muhammad Imran, Zhen-Lin Qiu, Ji Ma, Xinliang Feng

**Affiliations:** Department of Synthetic Materials and Functional Devices, Max Planck Institute of Microstructure Physics, Germany; Center for Advancing Electronics Dresden (cfaed) & Faculty of Chemistry and Food Chemistry, Technische Universität Dresden, Germany; Department of Synthetic Materials and Functional Devices, Max Planck Institute of Microstructure Physics, Germany; Center for Advancing Electronics Dresden (cfaed) & Faculty of Chemistry and Food Chemistry, Technische Universität Dresden, Germany; College of Materials Science and Opto-Electronic Technology & Center of Materials Science and Optoelectronics Engineering, University of Chinese Academy of Science, China; Department of Synthetic Materials and Functional Devices, Max Planck Institute of Microstructure Physics, Germany; Center for Advancing Electronics Dresden (cfaed) & Faculty of Chemistry and Food Chemistry, Technische Universität Dresden, Germany

## Abstract

This Perspective article outlines the design principles, key challenges, and future directions for advancing carbon-based molecular quantum technologies.

Molecular quantum materials provide a distinct chemical route toward next-generation quantum technologies, including quantum computing, secure information processing, non-volatile quantum memory and quantum sensing. Electron spins serve as powerful quantum resources, and their integration into nanocarbon molecular frameworks combines exceptional chemical tunability, structural diversity and synthetic scalability with the favorable spin physics required for quantum operations under ambient conditions (Fig. 1a). Although challenges remain in achieving long-term stability, scalable assembly and device integration, the chemical versatility of carbon nanostructures places synthetic organic/polymer chemistry at the forefront of next-generation quantum information science [1]. Here, we outline the design principles, key challenges and future directions for advancing carbon-based molecular quantum technologies.

**Figure 1.**
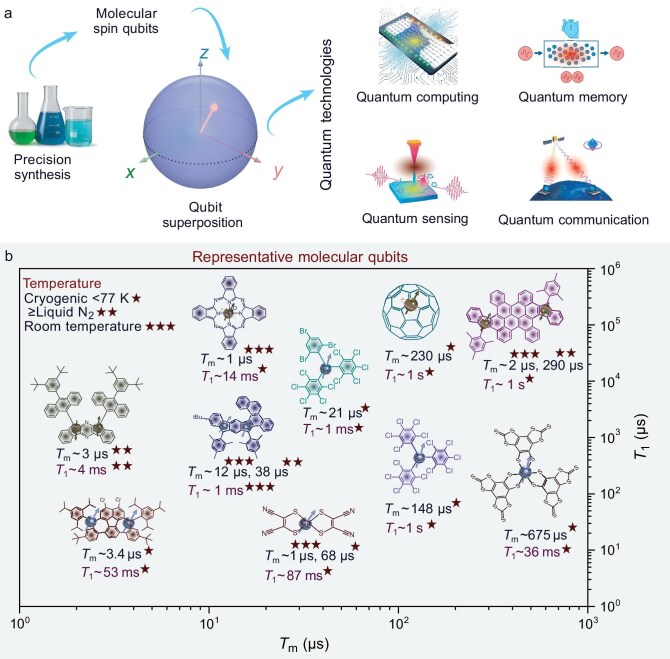
(a) Molecular spin qubits and their role in emerging quantum technologies. These chemically tunable molecular systems provide a bottom-up pathway toward quantum communication, computing, memory and sensing technologies. (b) Representative high-performance molecular qubit platforms, including endohedral fullerenes (N@C₆₀), transition-metal complexes, perchlorotriphenylmethyl (PTM) radicals and nanographene-based diradicaloids, illustrate how precision molecular synthesis enables control over spin states and coherent superposition. Notably, nanographene-based diradicaloids exhibit room-temperature long spin coherence times (*T*_m_) and spin–lattice relaxation time (*T*_1_) in the order of several microseconds, highlighting the potential of carbon-based molecular qubits for ambient quantum operation. Spin lifetimes are generally measured by pulsed EPR techniques and several measurement parameters critically affect both *T*_m_ and *T*_1_ such as pulse-sequence selection, matrix effects (proton-rich vs. deuterated), temperature and spin dilution. Most examples are taken from references provided and readers are directed to individual references for experimental details.

To date, several molecular platforms for spin qubits have been developed, including transition-metal complexes, endohedral fullerenes, organic radicals (e.g. trityl and nitroxides) and, more recently, nanographene-based radicaloids constructed from fused polycyclic aromatic rings. Across these systems, qubit performance is primarily evaluated by spin–lattice relaxation time (*T*_1_) and spin coherence time (*T*_m_), together with spectral addressability, environmental robustness and scalability toward coupled multi-spin architectures. Despite remarkable progress, including millisecond-scale coherence at cryogenic temperatures, achieving long *T*_m_ at or near ambient conditions remains a central challenge. Additional barriers include suppressing nuclear-spin and phonon-driven decoherence, ensuring chemical and spin-state stability under operation, enabling precise control over spin–spin couplings and realizing scalable device-level integration. In this context, carbon-based molecular qubits, constructed from light elements (C, H, O and N), intrinsically exhibit weak spin–orbit coupling, reduced hyperfine interactions and exceptional chemical tunability, thereby establishing them as promising qubit architectures compatible with ambient-condition quantum operations.

Molecular qubits based on transition-metal complexes have achieved long spin coherence times particularly at cryogenic temperatures through isotopic engineering to reduce nuclear-spin noise, rigid ligand environments to suppress spin–phonon coupling, and clock transitions that reduce sensitivity to magnetic-field fluctuations, thereby establishing important performance benchmarks for molecular quantum platforms [2]. In parallel, molecular qubits based on stable organic radicals including N-oxide and trityl-based radicals have emerged as important molecular qubits, with recent advances with TEMPO-based chromophores and luminescent tris(2,4,6–trichlorophenyl)methyl (TTM) diradicals demonstrating optical initialization and readout of high-spin states [3,4]. On the other hand, nanographene-based spin qubits combine high structural rigidity with electronic tunability and extended spin delocalization. These attributes directly suppress spin–spin and spin–lattice interactions, thus providing a promising route toward enhanced quantum coherence. Our collaborative work with Bogani’s group revealed that a nanographene-based diradicaloid could achieve a room-temperature *T*_m_ of ∼2 µs [5]. More recently, we have shown that carefully designed diradicaloids could reach *T*_m_ of ∼12 µs at room temperature and up to 38 µs at 200 K, representing some of the longest coherence times reported for carbon-based molecular spin systems near ambient conditions [6]. Together, these advances suggest that long-lived coherence in carbon-based molecular qubits is achievable at temperatures well above cryogenic levels (Fig. 1b).

Advancing carbon-based molecular quantum materials requires an integrated, multidisciplinary effort encompassing molecular design, computational modeling and the elucidation of robust structure–property relationships. The subsequent guidelines delineate the critical research directions that are poised to shape future progress in this field.

From a chemical design perspective, achieving a balance between chemical stability and sufficiently high diradical character is essential for robust molecular qubit behavior. Thermodynamic stabilization via extended π-delocalization stabilizes open-shell states and disperses spin density, helping suppress local decoherence channels. However, excessive delocalization may enhance spin–phonon coupling via π-stacking, underscoring the need to balance electronic and structural factors. Incorporating rigid, planar molecular frameworks suppresses low-frequency vibrational modes and spin–phonon coupling that drive decoherence, as demonstrated by rigidified TTM-based architectures featuring fused aryl units or acetylenic bridges, which exhibit extended spin coherence times [7]. Kinetic stabilization, typically introduced through sterically protective substituents, is equally critical for suppressing dimerization and chemical degradation; however, substituents must be chosen judiciously. Excessively flexible, proton-rich groups may introduce additional hyperfine and vibrational noise, whereas rigid, low-proton frameworks would enhance both stability and quantum coherence. In addition, constructing nuclear spin-free environments especially through systematic proton–deuterium substitution can effectively suppress hyperfine-mediated spin flip-flop processes, enabling the rational design of high-performance molecular qubits.

The chemical landscape for carbon-based molecular qubits is still largely unexplored, offering tremendous opportunity to design new open-shell carbon nanostructures and identify π-conjugated molecules that have yet to be evaluated as suitable qubit candidates. Systematic exploration of Kekulé vs. non–Kekulé structures, singlet–triplet gaps and degrees of electronic correlation will be essential for understanding how structural features govern spin coherence time and spin entanglement. Developing stable and synthetically accessible molecular qubits will provide testbeds for probing fundamental structure–spin property relationships. A predictive map linking molecular structure to quantum performance (e.g. *T*_1_ and *T*_m_), remains a major unmet need. Comparative studies will enable data-driven correlations between structure and quantum performance, providing the design rules needed to move the field from empirical discovery to rational, chemistry-guided qubit development.

Going beyond single-spin qubits to multi-radical and high-spin systems opens pathways toward qudits, quantum error correction and more compact quantum logic architectures. While recent on-surface synthesis of carbon-based multi-spin architectures has yielded valuable structural and spectroscopic insights into engineered spin arrays, these systems have largely been probed by scanning probe methods rather than time-domain coherent spin control. Achieving coherent quantum manipulation in such architectures therefore remains an open challenge [8]. Recent advances in 2D metal–organic frameworks and conjugated-polymer qubit platforms demonstrate that precise solid-state spin manipulation and high-fidelity coherent control are increasingly achievable [9,10]. Long-term visions for molecular quantum science include assembling ordered 2D qubit networks with tunable coupling, controlled spacing and coherent manipulation, an ambitious goal that demands both synthetic breakthroughs and technological advances for individual qubit addressability, such as scanning tunneling microscope electron paramagnetic resonance (EPR), frequency-selective driving or localized field control, akin to approaches used in neutral atom qubits. Such architectures could serve as programmable spin chains, spin lattices, quantum simulators or even prototype molecular-scale quantum processors.

Similarly, modern quantum-chemical methods are becoming indispensable for accelerating the discovery and optimization of molecular qubits. Computational techniques such as density functional theory continue to advance in their ability to evaluate frontier orbital energies, exchange coupling and spin-density delocalization in open-shell radicaloids, even though direct prediction of coherence times remains challenging. Recent developments in spin-dynamics theory now allow explicit modeling of spin–vibrational coupling through dynamic spin Hamiltonians, providing molecular-level insights into the dominant decoherence pathways of *S* = 1/2 molecular qubits [11]. Integrating these models with machine-learning approaches based on experimental datasets offers a powerful route towards rational design and optimization of high-coherence molecular qubits.

Lastly, reliable measurement of *T*_1_ and *T*_m_ values through pulsed EPR techniques remains critical. Pulse-sequence selection, matrix effects (proton-rich vs. deuterated), temperature-dependent behavior of spin states, and careful spin dilution all critically influence coherence time measurements. Establishing standardized pulsed EPR protocols across laboratories will be essential for enabling fair comparison, reproducibility and reliable modeling of coherence behavior [12].

Achieving room-temperature coherence times (*T*_m_) in the order of 100 μs in carbon-based molecular qubits, sufficient to support multi-gate quantum operations based on the ΩR*T*_m_ > 10⁴–10⁵ criterion, where ΩR is the Rabi frequency, would represent a transformative milestone [2]. Such a breakthrough would enable ambient-operating quantum prototypes, spin-based logic operations and potential integration with classical electronics. While the field is young, it is rich with opportunity, propelled by the growing creativity of synthetic chemists entering the quantum domain. Realizing the full potential of molecular qubits will depend on sustained, close collaboration that bridges chemistry, physics, materials science and computation. With continued advances in molecular spin chemistry and physics, ambient quantum technologies built from carbon-based molecular qubits appear increasingly within reach.
